# Supplementary material for: Ranking the effectiveness of non-pharmaceutical interventions to counter COVID-19 in UK universities with vaccinated population
Source: Sci Rep. 2022 Jul 29;12:13039. doi: 10.1038/s41598-022-16532-5 (PMC9336164; doi:10.1038/s41598-022-16532-5)
Supplement: Supplementary file 1 — Supplementary Information. [file 41598_2022_16532_MOESM1_ESM.pdf]

Supplementary Material - Ranking the Effectiveness of Non-Pharmaceutical Interventions to Counter COVID-19 in UK Universities with Vaccinated Population

Zirui Niu[1] and Giordano Scarciotti[1*]

[1]EEE, Imperial College London, Exhibition Rd, South Kensington, London, SW7 2AZ, United Kingdom.

*Corresponding author(s). E-mail(s): g.scarciotti@imperial.ac.uk;
Contributing authors: z.niu@outlook.com;

Appendix A Sensitivity Figures

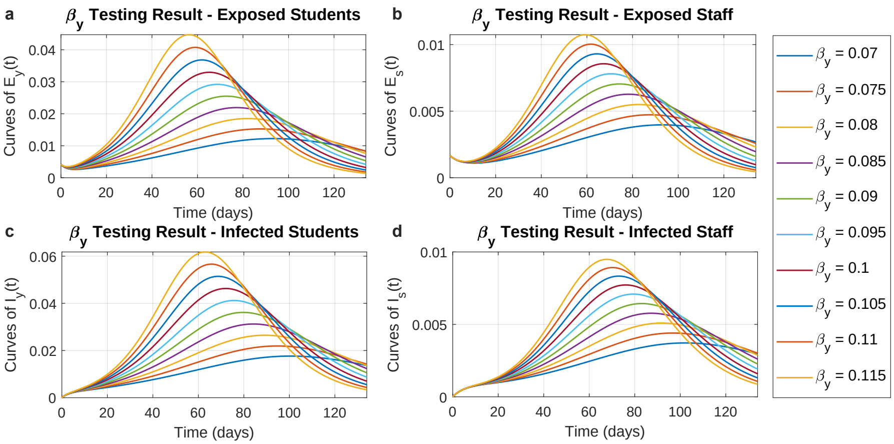

Fig. A1 Numerical testing of student-to-student infection rate β_y. Trajectories of cases among students and staffs when β_y varying from 0.07 to 0.115. With different β_y, panels **a** and **b** show the proportion trajectories of exposed students and staffs respectively while panels **c** and **d** depict the proportion trajectories of infected students and staffs respectively.

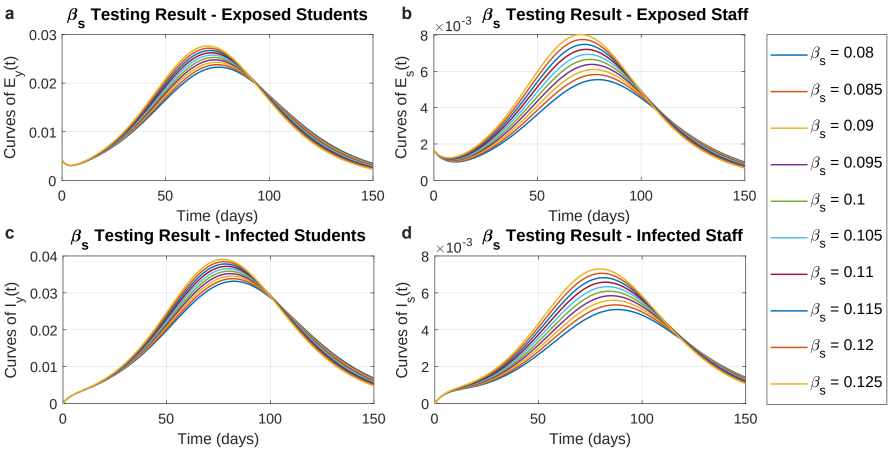

Fig. A2 Numerical testing of student-to-student infection rate β_s. Trajectories of cases among students and staffs when β_s varying from 0.08 to 0.125. With different β_s, panels **a** and **b** show the proportion trajectories of exposed students and staffs respectively while panels **c** and **d** depict the proportion trajectories of infected students and staffs respectively.

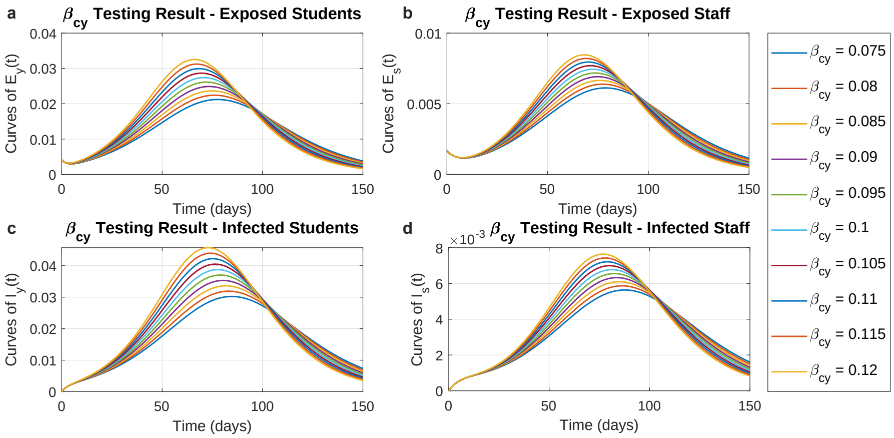

Fig. A3 Numerical testing of student-to-student infection rate β_{cy}. Trajectories of cases among students and staffs when β_{cy} varying from 0.075 to 0.12. With different β_{cy}, panels **a** and **b** show the proportion trajectories of exposed students and staffs respectively while panels **c** and **d** depict the proportion trajectories of infected students and staffs respectively.

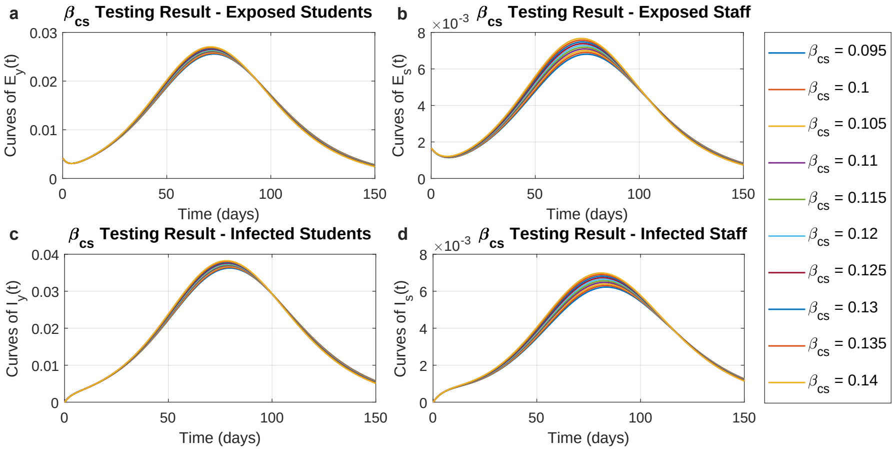

Fig. A4 Numerical testing of student-to-student infection rate β_{cs}. Trajectories of cases among students and staffs when β_{cs} varying from 0.095 to 0.14. With different β_{cs}, panels **a** and **b** show the proportion trajectories of exposed students and staffs respectively while panels **c** and **d** depict the proportion trajectories of infected students and staffs respectively.

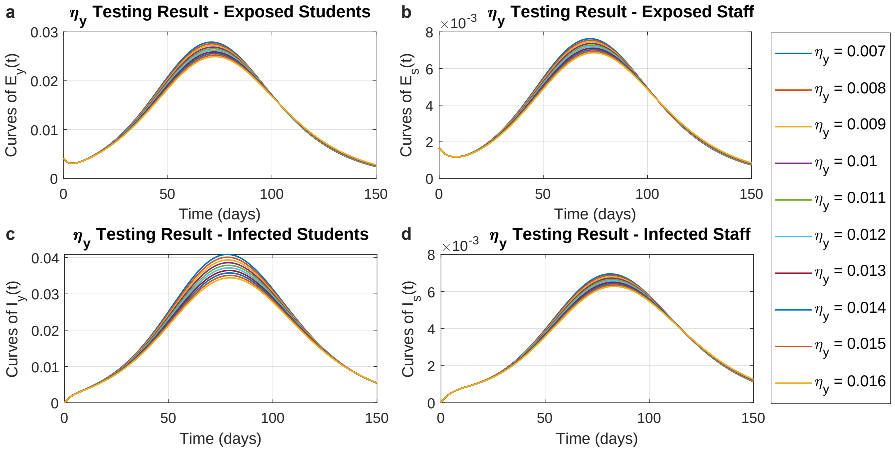

Fig. A5 Numerical testing of student-to-student infection rate η_y. Trajectories of cases among students and staffs when η_y varying from 0.007 to 0.016. With different η_y, panels **a** and **b** show the proportion trajectories of exposed students and staffs respectively while panels **c** and **d** depict the proportion trajectories of infected students and staffs respectively.

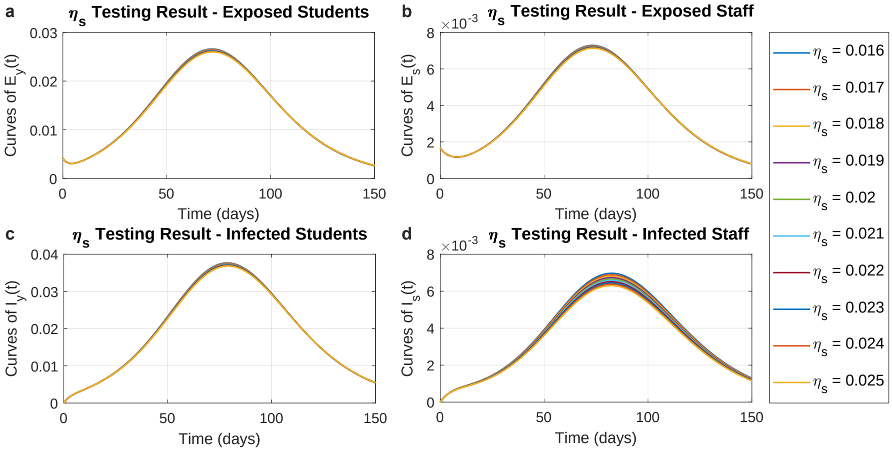

Fig. A6 Numerical testing of student-to-student infection rate $\boldsymbol{\eta_s}$. Trajectories of cases among students and staffs when η_s varying from 0.016 to 0.025. With different η_s, panels **a** and **b** show the proportion trajectories of exposed students and staffs respectively while panels **c** and **d** depict the proportion trajectories of infected students and staffs respectively.

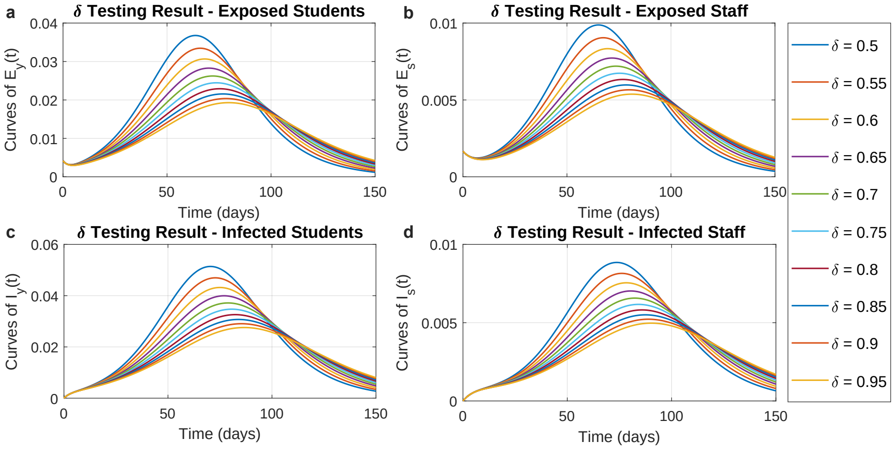

Fig. A7 Numerical testing of student-to-student infection rate $\boldsymbol{\delta}$. Trajectories of cases among students and staffs when δ varying from 0.5 to 0.95. With different δ, panels **a** and **b** show the proportion trajectories of exposed students and staffs respectively while panels **c** and **d** depict the proportion trajectories of infected students and staffs respectively.

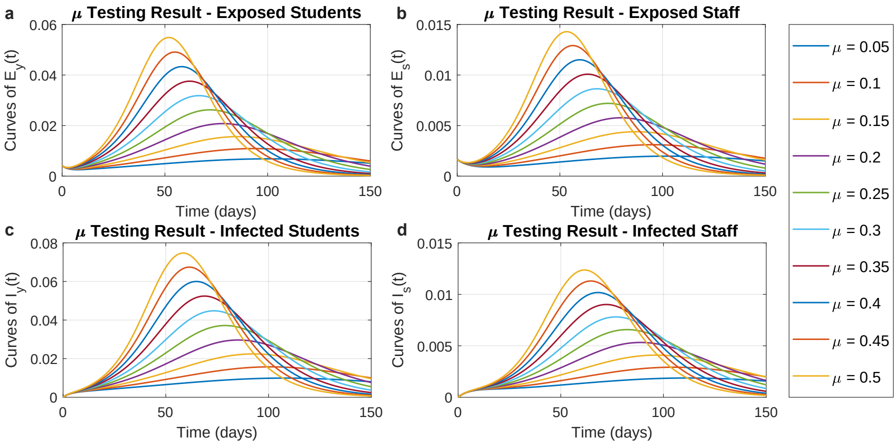

Fig. A8 Numerical testing of student-to-student infection rate μ. Trajectories of cases among students and staffs when μ varying from 0.05 to 0.5. With different μ, panels **a** and **b** show the proportion trajectories of exposed students and staffs respectively while panels **c** and **d** depict the proportion trajectories of infected students and staffs respectively.
